# Machine Learning Model Stability for Sub-Regional Classification of Barossa Valley Shiraz Wine Using A-TEEM Spectroscopy

**DOI:** 10.3390/foods13091376

**Published:** 2024-04-29

**Authors:** Han Wang, David W. Jeffery

**Affiliations:** School of Agriculture, Food and Wine, and Waite Research Institute, The University of Adelaide, PMB 1, Glen Osmond, SA 5064, Australia

**Keywords:** terroir, wine authentication, fluorescence spectroscopy, sub-regionalisation, artificial intelligence, *Vitis vinifera*

## Abstract

With a view to maintaining the reputation of wine-producing regions among consumers, minimising economic losses caused by wine fraud, and achieving the purpose of data-driven terroir classification, the use of an absorbance–transmission and fluorescence excitation–emission matrix (A-TEEM) technique has shown great potential based on the molecular fingerprinting of a sample. The effects of changes in wine composition due to ageing and the stability of A-TEEM models over time had not been addressed, however, and the classification of wine blends required investigation. Thus, A-TEEM data were combined with an extreme gradient boosting discriminant analysis (XGBDA) algorithm to build classification models based on a range of Shiraz research wines (n = 217) from five Barossa Valley sub-regions over four vintages that had aged in bottle for several years. This spectral fingerprinting and machine learning approach revealed a 100% class prediction accuracy based on cross-validation (CV) model results for vintage year and 98.8% for unknown sample prediction accuracy when splitting the wine samples into training and test sets to obtain the classification models. The modelling and prediction of sub-regional production area showed a class CV prediction accuracy of 99.5% and an unknown sample prediction accuracy of 93.8% when modelling with the split dataset. Inputting a sub-set of the current A-TEEM data into the models generated previously for these Barossa sub-region wines yielded a 100% accurate prediction of vintage year for 2018–2020 wines, 92% accuracy for sub-region for 2018 wines, and 91% accuracy for sub-region using 2021 wine spectral data that were not included in the original modelling. Satisfactory results were also obtained from the modelling and prediction of blended samples for the vintages and sub-regions, which is of significance when considering the practice of wine blending.

## 1. Introduction

Understanding the value of wine requires an appreciation of the influence of terroir—the interaction of physical, biological, and cultural aspects related to provenance and distinctive traits that influence product image, style, and quality. From its creation in the 1960s to today, the term terroir has endured and has even become the focus of investigations that aim to relate terroir to the properties of grapes and wine [1,2,3,4,5,6,7]. Considering its underpinnings, terroir necessarily encompasses research disciplines ranging from microbiology, plant and soil science, and oenology to marketing, consumers, humanities, and philosophy. Aside from the influence on grape and wine composition, the complex interactions contributing to terroir lead to a degree of recognition of wine produced in a certain (especially renowned) region, known as its ‘sense of place’ [8]. Despite the complexity and remaining scientific need to elaborate on the influence of terroir, wine producers endow terroir with commercial value and stimulate the potential institutional nature of terroir, making it a valuable marketing tool [1,9,10].

The institutionalisation of terroir has arisen because production regions are controlled according to Protected Designation of Origin (PDO) or Geographical Indication (GI) regulations, which aim to guarantee the authenticity and quality of a wine from a delimited region [1,8]. As reflected in the sub-regionalisation of wine regions, the subdivision of production zones has become a necessary means for the development of a wine-producing area because it is related to the reputation of the region and the interests of local enterprises [11,12,13]. This can be exemplified by the Barossa Valley, a typical Shiraz-producing region with a long history in Australia, which has stood at a historic turning point in the development of wine sub-regions. Five different potential sub-regions—Northern Grounds (NG), Central Grounds (CGs), Eastern Ridge (ER), Southern Grounds (SG), and Western Ridge (WR)—have been divided and are beginning to be recognised within the industry, becoming a tool to assist in the marketing of Barossa Valley wines [14,15].

In addition to the pursuit of quality, the sub-regionalisation of wine production regions is intended to be used as a means of wine marketing, thereby further increasing a wine’s value from a certain production area (with its associated terroir). This can be seen in the division of land into village appellations, which led to the rise in wine prices in Champagne and Burgundy [2,9,12,16]. Wine is thus viewed as a value-added luxury product and an important contributor to the global beverage market, with the European wine sector alone generating billions of dollars in revenue each year [17,18]. In this significant global market with substantial economic benefits, cases of profiteering through wine counterfeiting are common and have long plagued wine producers and local governments [19]. According to an EUIPO report in 2016, the existence of counterfeit wine in the European Union leads to an estimated annual revenue loss of about USD 1.3 billion, equivalent to 3.3% of total sales and an employment loss of about 4800 jobs [20]. As a mainstay of the global wine industry, Australia has also been affected by wine fraud, with counterfeit wine under the famous Penfolds brand, for example, flowing freely in overseas markets [21]. Within Australia, wine fraud more likely relates to honesty around wine label information, the underlying details of which could be altered during the winemaking stage in terms of vintages, varieties, and regional blends, especially in relation to the 85% blending principle [22].

As a wine authentication method, the absorbance–transmission and fluorescence excitation–emission matrix (A-TEEM) approach is based on absorbance and fluorescence spectroscopy [23,24] using an Aqualog instrument with right-angle optical geometry for fluorescence detection [25,26]. This method can generate multidimensional spectral information in the UV–Vis range for all chromophores and fluorophores and simultaneously combines absorbance–transmittance data with an excitation–emission matrix (EEM) to provide unique molecular fingerprints of wine [27]. The total EEM data obtained with this technology involve a set of emission spectra across different wavelengths (λ_em_), recorded within a range of excitation wavelengths (λ_ex_). This provides information on fluorescent substances in each wine sample and is effective for comparing samples with small compositional differences [27,28,29]. Ranaweera et al. [30] explored the use of A-TEEM for encapsulating the influence of biophysical and cultural factors associated with wine terroir by tracking wines through the winemaking process, and A-TEEM yielded impressive results for discriminating regions within a single GI [31]. In a study on the origin traceability and authenticity verification of Chinese wine, the combination of EEM and chemometrics was once again proven effective as a wine traceability technology [32]. In addition, compared with traditional wine analysis techniques like high-performance liquid chromatography (HPLC), specific natural isotope fractionation–nuclear magnetic resonance (SNIF-NMR), and isotope ratio mass spectrometry (IRMS) [33,34,35], acquiring A-TEEM data is relatively simple and accessible, especially for those working in winery laboratories who are already familiar with UV–Vis spectrophotometry.

In terms of spectral data analysis, principal component analysis (PCA) and parallel factor analysis (PARAFAC) are commonly used chemometric techniques. A-TEEM data can also be combined with a machine learning algorithm, such as extreme gradient boosting discrimination analysis (XGBDA), to classify wines from different vintages, varieties, and regions and even different sub-regions in Barossa Valley. Such chemometric methods demonstrate the ability to analyse nuanced EEM data. PCA and XGBDA can find patterns in datasets and classify samples based on similarities and differences in unsupervised and supervised modes, respectively. As an auxiliary technique, PARAFAC can effectively identify the type and concentration of fluorophores underpinning a dataset classification [24,26,27,31].

Previous work has undoubtedly provided encouraging results for terroir classification at the sub-regional level, with the discrimination of close geographical regions being difficult to accomplish with other analytical methods [31]. The present study aimed to further explore the ultimate depth that this method can reach and consider scenarios encountered in the practical application of the method in the industry. The Barossa Valley GI remained the target region, with an additional vintage of 2021 added along with an assessment of stored wines used in the previous study [31], to explore the influence of bottle ageing. XGBDA was used for machine learning classification modelling, including for the prediction of unknown samples using models developed with a split dataset. The newly collected sample data for the stored wines were assessed with the prediction model established by Ranaweera, Bastian, Gilmore, Capone, and Jeffery [31] two years prior to determine how relevant that previous model was for classifying the current dataset. In addition, samples from the four vintages and five sub-regions were mixed in certain proportions to investigate the practice of wine blending, thereby providing relevance to a typical winemaking scenario.

## 2. Materials and Methods

### 2.1. Chemicals

High-purity water was obtained with the Milli-Q purification system (Elga Labwater, Woodridge, USA). Absolute ethanol for chromatography and analytical-grade hydrochloric acid (HCl, 37% *w*/*v*) were purchased from Rowe Scientific (Lonsdale, SA, Australia).

### 2.2. Wine Samples

Shiraz research wines (n = 217) produced in 2018, 2019, 2020, and 2021 from fruit collected in 20 vineyards were available from a previous project that investigated Barossa Shiraz terroir [36]. As reported before, the wines were made with 100% single-site Shiraz grapes, with fruit parcels obtained from four sites within each of the five sub-regions of Barossa Valley, South Australia, across the four vintages, as follows: Sites 1–4, Northern Grounds (NG) = 42 wines; Sites 5-8, Central Grounds (CG) = 36; Sites 9–12, Eastern Edge (ER) = 48; Sites 13–16, Southern Grounds (SG) = 45; Sites 17–20, Western Ridge (WR) = 46. Replicate wines were available for each site (A, B, C), as shown in Table S1 of the Supporting Information. Winemaking was undertaken by WIC Winemaking Services and wines were bottled in 750 mL glass bottles with screw caps. Bottled wines were stored in a wine cellar under controlled temperature and humidity. Wines from 2018–2020 analysed in the present work (excluding Eden Valley given the focus on Barossa Valley) had aged for an additional 2 years since their previous A-TEEM analysis reported by Ranaweera, Bastian, Gilmore, Capone, and Jeffery [31] (total ageing time of 3–5 years), whereas 2021 wines had been cellared for only 2 years and were analysed for the first time.

### 2.3. Sample Preparation and A-TEEM Procedure

Wine samples (1 mL) obtained from freshly opened bottles were centrifuged (Eppendorf 5415D, Adelab Scientific, Thebarton, SA, Australia) at 9300× *g* for 10 min. The supernatant (40 μL) was obtained and diluted by 1:150 with degassed and filtered (0.45 μm PTFE membrane) 50% aqueous ethanol adjusted to pH 2 with HCl, according to Ranaweera, Gilmore, Capone, Bastian, and Jeffery [23]. After dilution, samples were mixed with a benchtop vortexer (MS1 Minishaker IKA) for 60 s and sonicated for 15 min (SONICLEAN 250HD, Rowe Scientific, Lonsdale, SA, Australia) to remove air bubbles. Samples were analysed in duplicate using Hellma type 1FL (1 cm path length) macroscopic fluorescence cuvettes (Sigma-Aldrich, Castle Hill, NSW, Australia) using an Aqualog spectrophotometer (UV-800-C, HORIBA Scientific, Quark Photonics, Adelaide, SA, Australia). The settings consisted of 0.2 s integration time, excitation wavelength range 240–800 nm in 5 nm increments, emission range 242–824 nm in 4.66 nm increments, saturation mask width 10 nm, medium detector gain, and automatic spectral pre-processing including the correction of inner filter effects and Rayleigh masking. The EEMs were normalised by the measurement of a standard, sealed, high-purity water cuvette each time the instrument was used, as previously reported [26,31]. The diluted wine sample was stirred in the 1FL cuvette within the sample holder for 120 s with a stir bar before the start of the analysis. Dilution solvent blanks were recorded in the same way prior to sample analysis for auto-subtraction from each sample in the batch [37]. Absorption spectra (240–700 nm) and EEMs were recorded using Aqualog software (version 4.3, HORIBA Scientific, Quark Photonics).

### 2.4. Preparation of Wine Blends

Wines were prepared in a 12 mL glass vial with silicone/PTFE screw cap (Agilent Technologies, Santa Clara, CA, USA), following the mixture proportions shown in Tables S2–S4 to obtain a final volume of 10 mL of mixed wine sample (in duplicate). The approach was similar to that reported by Ranaweera et al. (2022). After thoroughly mixing vials for 60 s using a benchtop vortex, samples were prepared as above (i.e., centrifuged at 9300× *g* for 10 min in a 10 mL centrifuge tube; 40 μL of supernatant diluted 150-fold with dilution solvent; samples vortexed to mix and finally sonicated) and analysed to obtain A-TEEM data.

### 2.5. Statistical Analysis

SOLO + MIA (version 9.2.1.0, Eigenvector Research, Inc., Manson, WA, USA) was used for data processing and analysis.

#### 2.5.1. Data Fusion (Multi-Block Modelling)

The 3D EEMs and corresponding 2D absorbance datasets from A-TEEM were combined to enhance the classification and prediction accuracy [24]. Then, 3D EEM data were reshaped into a two-way data array (unfold multiway mode 1) and joined with the absorbance data. Fused data were used in statistical analyses requiring a 2D dataset, namely, PCA and extreme gradient boosting (XGBoost) modelling.

#### 2.5.2. Unsupervised Data Analysis

PCA and PARAFAC were variously applied to analyse the different datasets collected using the A-TEEM method. For PCA, fused data were auto-scale pre-processed with five principal components selected to classify the five different sub-regions for each of the four vintages. PARAFAC was used to decompose the 3D EEM data of the wine samples into the most dominant fluorophores. For pre-processing, normalisation of spectra to 1 (default) and EEM filtering were applied, with ±16 nm and ±32 nm for the first-order and second-order Rayleigh filters, respectively [38]. Non-negativity constraints were imposed in all three modes (intensity, emission, and excitation wavelengths) of EEM data, and components were selected based split-half analysis results [38].

#### 2.5.3. Data Analysis with Machine Learning

XGBDA was applied as a classification machine learning algorithm to build the wine authentication models with the fused dataset according to vintage, sub-region, and the specified blends. XGBDA was applied with partial least squares (PLS) compression, using a maximum for latent variables (LVs) of 10 for vintage and vintage blending classification and 20 LVs for sub-region and sub-region blending classification (blends as specified in Table S2 of the Supporting Information). The models developed for vintage and sub-region were applied to the blends specified in Tables S3 and S4, respectively. The number of LVs was selected according to the cross-validation (CV) result accuracy when comparing 10–45 LVs. Pre-processing was undertaken with mean centring, autoscaling, and generalised least squares weighting (GLSW) with the declutter threshold at 0.02 to calibrate and cross-validate (Venetian blinds procedure, k = 10). The xgboost algorithm and gbtree booster of XGBDA had an eta = 0.3, max_depth = 1, and num_round = 200. Model testing for both vintage and sub-region was taken further by splitting the data into about 80% used for calibration (n = 354) and about 20% used for validation (n = 80) (keeping the replicates together), using the same XGBDA approach as just described. Further validation was obtained by loading a random subset of the multi-block sample data obtained in the present work (for vintage based on 2018–2020 and for sub-region using 2018 and 2021 as examples) into the previously established model (based on the combination of vintage and sub-region) [31] to test prediction accuracy with newly recorded data for the wines that had aged for a further 2 years and for 2021 wines that were not used before in the classification modelling. 

According to the most probable prediction rule, which assigns samples to the class with the highest probability overall, the validity of the model’s prediction results was evaluated by the confusion matrix score probability. The scoring probabilities included true positive (TP), false positive (FP), true negative (TN), and false negative (FN). The magnitude of the probability was expressed as a number from 0 to 1 and a percentage.

## 3. Results and Discussion

### 3.1. Molecular Fingerprints (EEMs)

**Figure 1:**
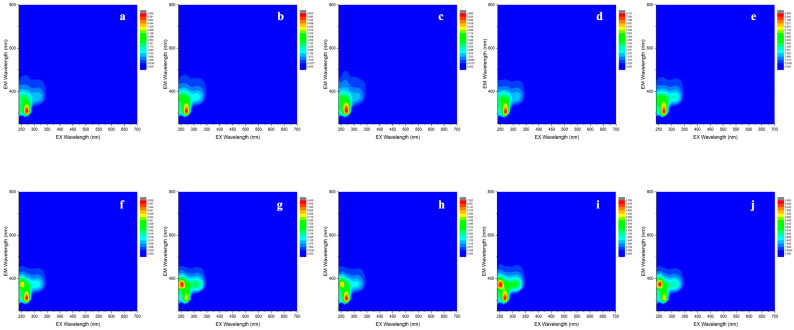
Fluorescence excitation and emission matrix (EEM) contour plots (molecular fingerprints) of Shiraz wines, comparing vintage 2018 for subregions (**a**) Northern Grounds, (**b**) Central Grounds, (**c**) Eastern Ridge, (**d**) Southern Grounds, and (**e**) Western Ridge and vintage 2021 for subregions (**f**) Northern Grounds, (**g**) Central Grounds, (**h**) Eastern Ridge, (**i**) Southern Grounds, and (**j**) Western Ridge.

Figure 1 shows examples of molecular fingerprints for experimental Shiraz wines from Barossa Valley GI, indicating the variance between the 2018 and 2021 vintages for the five sub-regions (NG, CG, ER, SG, WR). The vintage difference can primarily be seen through the gross differences in the EEM fingerprints. Each panel in the first row from vintage 2018 (Figure 1a–e) had only one intense peak at around λ_ex_/λ_em_ 270/310 nm, with panels in the second row from vintage 2021 (Figure 1f–j) having two intense peaks with λ_ex_/λ_em_ at around 270/310 nm and 250/370 nm. Comparing the fingerprints of vintage 2021 wines, Northern Grounds (Figure 1f) and Eastern Ridge (Figure 1h) tended to have a similar fingerprint, as did Central Grounds (Figure 1g) and Western Ridge (Figure 1j), whereas Southern Grounds (Figure 1i) had a more unique fingerprint. In contrast, spectra for the 2018 wines (5 years old) were more similar across the sub-regions. The differences between vintage 2018 and 2021 and sub-region differences within vintage 2021 could be explained on the basis of climatic data such as growing season rainfall, mean January temperature, and growing degree days, as well as other terroir influences [31,39].

The EEMs were generally representative of the spectral fingerprints obtained with the A-TEEM approach, and, as seen with the sub-regions for vintage 2018, differences may not have been easily discernible by simple visual inspection. This demonstrates the importance of using chemometrics with these datasets to identify subtle patterns in the EEM fingerprints, as elaborated in subsequent sections.

### 3.2. PARAFAC Decomposition of EEMs

**Figure 2:**
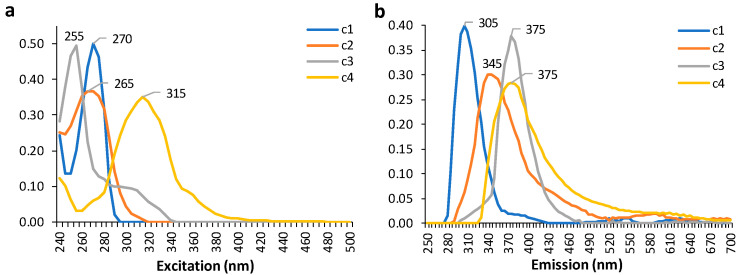
Loadings from parallel factor analysis (PARAFAC) decomposition modelling of 3D EEM data for Shiraz wine samples from all sub-regions and vintages showing (**a**) excitation wavelengths (nm) and (**b**) emission wavelengths (nm) of components 1–4.

**Figure 3:**
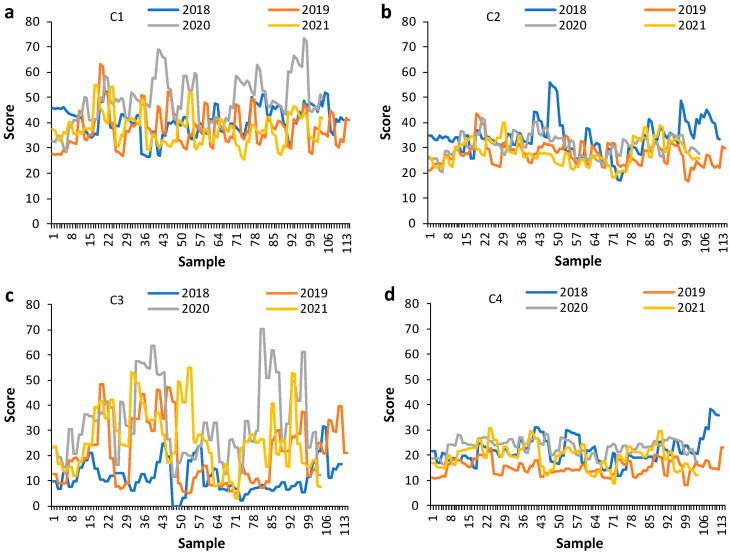
PARAFAC score plots for samples from different sub-regions according to vintages 2018–2021 for (**a**) component 1, (**b**) component 2, (**c**) component 3, and (**d**) component 4.

PARAFAC was undertaken to tentatively identify the main fluorophores that characterised the samples. These results could then be used to provide some understanding of the possible compositional drivers underpinning sub-regional classification. A four-component model comprising all sub-regions and vintage years was selected based on a split-half analysis of 97%. PARAFAC modelling (Figure 2) yielded a component 1 peak at 270/305 nm (λ_ex_/λ_em_), component 2 peak at 265/345 nm, component 3 peak at 255/375 nm, and component 4 peak at 315/375 nm. Components were tentatively assigned to respective compound classes: 1. flavan-3-ols [28,40]; 2. anthocyanins, aromatic amino acids, and hydroxybenzoic acids [28,40,41]; 3. phenolic acids/aldehydes and flavonols [28]; 4. caffeic and *p*-coumaric acids [40] and stilbenes like resveratrol and *trans*-piceid [41,42] or perhaps grape seed oils from maceration during fermentation (e.g., tocopherols and tetraenes) [43]. The PARAFAC score plots (Figure 3a–d) revealed how the vintages differed based on the tentatively assigned fluorophores.

The PARAFAC components related to ordinary red wine constituents that are influenced by grape growing conditions and terroir more broadly, which could be variable among the sub-regions used in this study. Components 2 (anthocyanins, amino and hydroxybenzoic acids) and 4 (hydroxycinnamates, stilbenes) showed less fluctuation according to vintage than components 1 (flavan-3-ols) and 3 (phenolic acids, flavonols). The seasonal climate could be a particular factor contributing to the variability in some vintage years more than others, with differences in growing season rainfall and temperature for 2018–2021 according to the vintage reports from Barossa Australia [44]. As noted earlier, the gross differences observed in the EEMs presented in Figure 1 that underpin the PARAFAC results could also be related to this observation. Relative wine age could also exert some influence based on the evolution of phenolic profiles over time. Depending on vintage year, greater differences among the sub-regions were also evident, especially for components 1 and 3 (Figure 3a,c).

### 3.3. PCA Decomposition of A-TEEM Data

**Figure 4:**
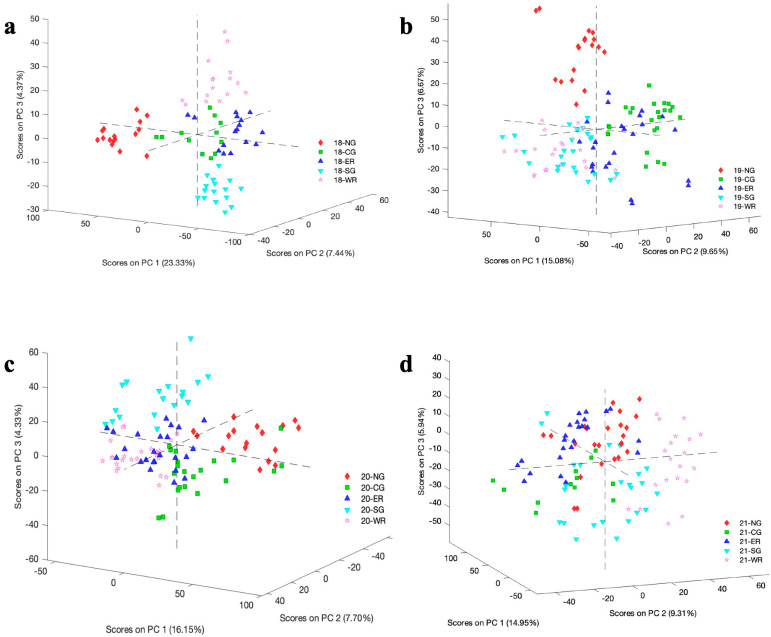
Principal component analysis score plots showing the first three principal components (PC 1, PC 2, PC 3) from multi-block wine sample data for five sub-regions across vintages (**a**) 2018, (**b**) 2019, (**c**) 2020, and (**d**) 2021. NG, Northern Grounds (red diamonds); CG, Central Grounds (green squares); ER, Eastern Ridge (dark blue triangles); SG, Southern Grounds (light blue inverted triangles); WR, Western Ridge (lilac stars).

Dimensionality reduction with PCA was applied to multi-block A-TEEM data (i.e., combined absorbance and EEM datasets) to explore the separation of Barossa sub-regions (Figure 4). The first three principal components accounted for a total variance explained of 35.1%, 31.4%, 28.2%, and 30.2% for vintages from 2018 to 2021, respectively. Vintage 2018 in particular showed an impressive result, with each sub-region tightly grouped and almost completely separated from each other (Figure 4a). This was reminiscent of the results obtained for this vintage in the previous work [31]. However, apart from NG (red diamonds) in 2019 (Figure 4b), and 2020 to a lesser extent (Figure 4c), the other vintages did not exhibit significant differentiation of sub-regions according to PCA. SG (light blue inverted triangles) and WR (lilac stars) were similar to each other in vintages 2019–2021, whereas NG/CG/SG showed an obvious degree of separation in the four vintages, although less so in 2021 (Figure 4d), especially for NG and CG (green squares). The separation of WR and ER (dark blue triangles) from the other three sub-regions largely depended on the vintage, and WR and ER were themselves separated to a degree in vintages 2018 and 2021 (Figure 4a,d). These results were consistent with the study published by Ranaweera, Bastian, Gilmore, Capone, and Jeffery [31]: besides climate factors across vintages (and regions) as mentioned in previous sections, which could lead to more or less differentiation, localised factors of terroir such as soil properties and topography across sub-regions could play a role [15,39] via their influences on grape (and, thus, wine) composition.

Ageing could be another factor that correlated with the separation of sub-regions in the PCA plot (greater separation for older wines), although vintage differences might have a more pronounced influence, considering that the separation of sub-regions for 2018–2020 wines was more or less maintained upon re-analysis of the wines after several years of bottle ageing. This is an important result from an implementation perspective—despite the compositional changes associated with red wines as they age, which can impart changes in wine EEM fingerprints and absorbance values, the original differentiation among the sub-regions according to PCA was still evident several years later. Even so, PCA with multi-block spectral data for these aged wines from different vintages was not sufficient to consistently separate the sub-regions, although k-means clustering was able to resolve vintage year in the previous work [31]. Improvement in sub-regional classification across multiple vintages was necessary, with supervised methods and particularly machine learning algorithms providing a possible solution, as evidenced previously [24,31].

### 3.4. XGBDA Predictive Modelling

#### 3.4.1. Vintage and Sub-Region Validation

**Figure 5:**
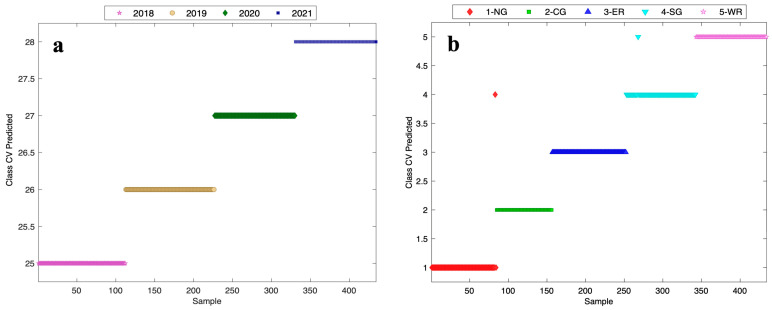
Class predicted from cross-validation (CV) using extreme gradient boosting discriminant analysis (XGBDA) modelling of multi-block A-TEEM data according to (**a**) vintage and (**b**) sub-region. Wine samples (n = 217 in duplicate) from four vintages in (**a**) 2018 (lilac stars), 2019 (yellow circles), 2020 (green diamonds), and 2021 (blue squares) and five sub-regions in (**b**) NG (red diamonds), CG (green squares), ER (dark blue triangles), SG (light blue inverted triangles), and WR (lilac stars).

As an effective machine learning classification algorithm [23,30], XGBDA was carried out in an attempt to improve classification across the vintages and sub-regions. The XGBDA approach with CV afforded an excellent classification result (Figure 5 and Table S5 of the Supporting Information), with 100% accuracy for the vintage model (Figure 5a), and 99.5% accuracy (2 misclassified out of 434 sample spectra, Figure 5b)) for the sub-region model. These exemplary results were consistent with the work of Ranaweera, Bastian, Gilmore, Capone, and Jeffery [31] and remarkable, considering the proximity of the sub-regions. A further step of splitting the datasets into about 80% for calibration (n = 354) and about 20% for validation (n = 80) led to slightly lower accuracy than the CV model results shown in Figure 5: in this case, 1 out of 80 samples was misclassified for vintage, giving a 98.8% classification accuracy (Figure S1a–d of the Supporting Information), and 5 out of 80 samples were misclassified at a sub-region level, giving a 93.8% classification accuracy (Figure S2a–e). Ideally, greater sample numbers would be used for splitting the datasets, but this outcome still highlights the robustness of the A-TEEM approach for classifying wine samples, with the accuracy easily being equal to other authentication techniques. Again, though, it is worth remarking that this study considered wines from sub-regions within a GI (as little as several km apart), thus highlighting the ability to authenticate at a fine scale and the potential of the approach for helping to objectively define unique terroirs within regions.

Furthermore, in view of the possible effects of bottle ageing on the A-TEEM data mentioned earlier, and to obtain a deeper understanding of the impact of bottle ageing on this authentication method, the multi-block A-TEEM data obtained in the present study were tested against the previous model developed by Ranaweera, Bastian, Gilmore, Capone, and Jeffery [31] using a subset of the same wine samples from 2018–2020, as well as wines from vintage 2021, which were not analysed previously. According to Figure S3a of the Supporting Information, the prediction of vintage for 2018, 2019, and 2020 wines with the previous model still showed 100% classification accuracy. For the prediction of sub-region, highlighted for 2018 and 2021 wines (excluding Eden Valley, which was not analysed in the present work), the model not only achieved prediction of the 2018 vintage samples with an accuracy of 92% (Figure S3b, four misclassified samples) but it also achieved a prediction accuracy of 91.2% for the samples from vintage 2021 (Figure S3c, three misclassified samples), which had not been involved in the development of the previous model. This was a highly encouraging result as it not only meant that the method was largely unaffected by bottle ageing (i.e., wines measured several years later could still be accurately predicted with the originally developed models) but also demonstrated the sub-region predictive ability using data from an ‘unknown’ vintage (i.e., wines from 2021).

#### 3.4.2. Blended Wine Validation

Considering that commercial wines typically consist of blends, it was worthwhile considering the performance of the A-TEEM and XGBDA classification method upon wine blending. Previously, wines were tracked through the winemaking process and XGBoost regression (XGBR) modelling highlighted the sensitivity of the approach to blending one varietal wine with as little as 1% of another [30]. As an extension, selected samples in the present study were blended across different vintages and separately for different sub-regions. The blending ratio for sub-regions was set as 50:50 and also 15:85, according to the Australian wine industry 85% principle [22], as well as 50:50, 10:90, and 5:95 for vintage (Tables S2–S4 of the Supporting Information).

**Figure 6:**
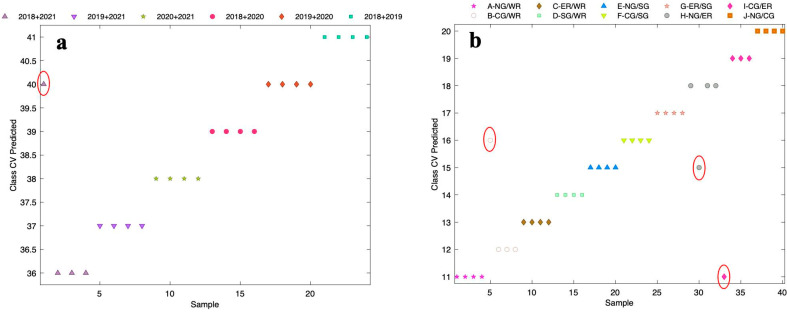
Class CV predicted from XGBDA modelling of multi-block A-TEEM data for different blends (50:50, prepared as outlined in Table S2) according to (**a**) vintage and (**b**) sub-region. Samples were blended in duplicate, and each was analysed in duplicate. NG, Northern Grounds; CG, Central Grounds; ER, Eastern Ridge; SG, Southern Grounds; WR, Western Ridge. Samples outlined in red were misclassified.

As the first step, multi-block A-TEEM data of 50:50 mixed samples (Table S2) were selected to establish the model and explore the predictive power of XGBDA with CV. Figure 6a shows the class CV predicted results of 12 blended samples (analysed in duplicate) from combinations of the four vintages, with only one wine misclassified and achieving 95.8% overall classification accuracy (Table S5); the combination of 2018 + 2021 was classified as 2019 + 2020. Figure 6b shows the class CV predicted results of 20 samples (analysed in duplicate) from blending combinations of the five sub-regions, showing that three samples were misclassified (92.5% accuracy, Table S5): one from CG + WR was classified as CG + SG, one from NG + ER was classified as NG + SG, and one from CG + ER was classified as NG + WR. Despite the limited selection of data, the results were consistently quite outstanding for both vintage and sub-region blending.

A further step was carried out by using the XGBDA models established in Section 3.4.1 of the Results and Discussion with multi-block A-TEEM data from selected samples (prepared in duplicate for a single analysis of each) using a stricter blending ratio of 10:90 and 5:95 for vintages (Table S3 of the Supporting Information), along with 15:85 and 50:50 for sub-regions (Table S4), to predict the probable class of each. Table 1 shows the prediction probability based on the vintage and sub-region blending. For wine samples comprising 95% vintage 2018 and 5% vintage 2021 wines (S1 and S2 for vintage), applying the model developed for vintage for all wines gave an average probability of 97.5% that the wine was from the 2018 and a 1.2% probability it was from 2021. For blends containing 90% 2018 wine and 10% 2021 wine (S3 and S4 for vintage), the model predicted that the samples were from 2018 and 2021 with averages of 89.95% and 8.75% probability, respectively. One sample containing 85% SG and 15% WR (S1 for sub-region) was predicted to consist of SG and WR wine with 89% and 6% probability, respectively, but the prediction of S2 for sub-region was extremely low, with only 6.9% and 1.1% probability of the sample coming from SG and WR, respectively. This was an anomalous result without an apparent explanation. Blends of 50% SG and 50% WR (S3 and S4 for sub-region, Table 1) were predicted to come from SG and WR with averages of 49.8% and 33.3% probability, respectively. Although these results did not automatically imply that class prediction should equal the percentage in the blend, the modelling did reasonably well (errant result aside) in reflecting the main vintage or sub-region component where one predominates, and at least indicates that any blend was not mistaken as a single vintage or individual sub-region.

The results presented in Table 1 nicely supplement the work of Ranaweera, Gilmore, Bastian, Capone, and Jeffery [30], who reported the use of XGBR modelling of the percentage of grape variety in a blend, with the present study addressing some gaps related to vintage and sub-region blending using A-TEEM data and XGBDA. Importantly, the results in Table 1 were obtained by loading blended wine sample data into the models established using the entire wine datasets for vintage and sub-region that did not involve any blends, thus providing further insight into the potential for the application of this wine authentication methodology in an industry-relevant context.

## 4. Conclusions

Overall, reliable results have been obtained for the classification of Shiraz research wines arising from adjacent areas within the Barossa Valley GI. The capability of A-TEEM and XGBDA has again shown its worth, with the important contribution of identifying subtle differences among wine samples after a period of bottle ageing but still being able to accurately classify such wines. This adds further weight to the utility of this approach regarding the stability of models over time, which is critical from a classification perspective as wines age. Notably, there was discernment of closely located vineyards even after wine ageing, thus highlighting the conservation of terroir influences on the spectral fingerprints of the wines. The ability of the technique to differentiate wine blends was also an important development in this work, considering that the blending of wine is a widespread and often necessary practice, but one that can be open to manipulation (through the falsification of region or variety, for example). Future improvements on the present outcomes can be envisaged by increasing the size of the dataset or, indeed, creating models for specific blends (potentially allowing for the detection of an unauthorised variety of a PDO wine). In addition, research could be extended to the analysis of commercial wines and the development of an authentication database over numerous vintages based on A-TEEM with machine learning classification.

## Figures and Tables

**Table 1:** Class-predicted probability from XGBDA modelling of multi-block A-TEEM data for blended samples based on vintage (2018 and 2021, prepared as outlined in Table S3) and sub-region (Southern Grounds and Western Ridge, prepared as outlined in Table S4).

	**Vintage**					
	**2018**			**2021**		
**Sample**	**Actual**	**Predicted**	**Relative Difference**	**Actual**	**Predicted**	**Relative Difference**
S1	95%	97.30%	2.4%	5%	1.30%	−74%
S2	95%	97.80%	2.9%	5%	1.10%	−78%
S3	90%	89.10%	−1.0%	10%	6.30%	−37%
S4	90%	90.80%	0.9%	10%	4.90%	−51%
	**Sub-Region**					
	**Southern Grounds (SG)**			**Western Ridge (WR)**		
**Sample**	**Actual**	**Predicted**	**Relative Difference**	**Actual**	**Predicted**	**Relative Difference**
S1	85%	88.50%	4.1%	15%	6.20%	−59%
S2	85%	6.90%	−91.9%	15%	1.10%	−93%
S3	50%	41.00%	−18.0%	50%	32.20%	−36%
S4	50%	58.60%	17.2%	50%	34.50%	−31%

## Data Availability

The original contributions presented in the study are included in the article/Supplementary Materials; further inquiries can be directed to the corresponding author.

## Supplementary Materials

The following supporting information can be downloaded at https://www.mdpi.com/article/10.3390/foods13091376/s1: Table S1: Summary of the different vintages, sub-regions, and sites of the Shiraz wines from the Barossa Valley GI analysed in this study; Table S2: Combinations of wine samples in blending (50:50) for A-TEEM analysis with 6 groups for vintage blending and 10 groups for sub-region blending; Table S3: Percentage of wine in the blend of 2018 and 2021 vintages; Table S4: Percentage of wine in the blend of Southern Grounds (SG) and Western Ridge (WR) sub-regions; Table S5: Confusion matrices showing the performance parameters of different cross-validated XGBDA models from multi-block data; Figure S1: Class-predicted member for test set samples (n = 80) from XGBDA modelling of multi-block A-TEEM data for vintage; Figure S2: Class-predicted member for test set samples (n = 80) from XGBDA modelling of multi-block A-TEEM data for subregion; Figure S3: Class-predicted member using the XGBDA model established by Ranaweera et al. (2023) with multi-block A-TEEM test data from the present study [31].
