# Supplementary material for: Machine Learning Model Stability for Sub-Regional Classification of Barossa Valley Shiraz Wine Using A-TEEM Spectroscopy
Source: Foods. 2024 Apr 29;13(9):1376. doi: 10.3390/foods13091376 (PMC11083604; doi:10.3390/foods13091376)
Supplement: Supplementary file 1 [file foods-13-01376-s001.zip › foods-2961835-supplementary.pdf]

SUPPLEMENTARY MATERIAL FOR

Machine learning model stability for sub-regional classification of Barossa Valley Shiraz wine using A-TEEM spectroscopy

Han Wang and David W. Jeffery *

School of Agriculture, Food and Wine, and Waite Research Institute, The University of Adelaide, PMB 1, Glen Osmond, SA 5064, Australia

* Correspondence: david.jeffery@adelaide.edu.au

Table of Contents

	Page
**Table S1.** Summary of the different vintages, sub-regions, and sites of the Shiraz wines from the Barossa Valley GI analysed in this study, with total sample number for each category. Wines were produced in triplicate (indicated by A, B, C) for each site.	**S2**
**Table S2.** Combinations of wine samples in blending (50:50) for A-TEEM analysis with 6 groups for vintage blending and 10 groups for sub-region blending of Northern Grounds (NG), Central Grounds (CG), Eastern Ridge (ER), and Southern Grounds (SG). S1–4 etc. indicate sample numbers for blends that were prepared in duplicate, with each analysed in duplicate (i.e., 4 spectra were recorded per sample).	**S3**
**Table S3.** Percentage of wine in the blend of 2018 and 2021 vintages. Blended wine samples prepared in duplicate (i.e., S1 and S2, S3 and S4).	**S3**
**Table S4.** Percentage of wine in the blend of Southern Grounds (SG) and Western Ridge (WR) sub-regions. Blended wine samples were prepared in duplicate (i.e., S1 and S2, S3 and S4).	**S3**
**Table S5.** Confusion matrices showing the performance parameters of different cross-validated XGBDA models from multi-block data (EEM and absorbance) for (A) vintages (Figure 5a in the main paper), (B) subregions (Figure 5b), (C) blending between vintages (Figure 6a) and (D) blending between subregions (Figure 6b).	**S4**
**Figure S1.** Class predicted member for test set samples (n = 80) from XGBDA modelling of multi-block A-TEEM data for vintage (a) 2018 (lilac), (b) 2019 (yellow), (c) 2020 (green), and (d) 2021 (blue).	**S5**
**Figure S2:** Class predicted member for test set samples (n = 80) from XGBDA modelling of multi-block A-TEEM data for subregion (a) Northern Grounds (NG), (b) Central Grounds (CG), (c) Southern Grounds (SG), (d) Eastern Ridge (ER) and (e) Western Ridge (WR).	**S6**
**Figure S3.** Class predicted member using the XGBDA model established by Ranaweera et al. (2023) with multi-block A-TEEM test data from the present study showing vintage (a) 2018 (red), 2019 (green), and 2020 (blue), and the five sub-regions for vintage (b) 2018 and (c) 2021.	**S7**

Table S1. Summary of the different vintages, sub-regions, and sites of the Shiraz wines from the Barossa Valley GI analysed in this study, with total sample number for each category. Wines were produced in triplicate (indicated by A, B, C) for each site. [1]

No.	2018			2019			2020			2021			Sub-region	TOTAL
SR1	A	B	C	A	B	C	A	B	C	A	B	C	Northern Grounds (NG)	42
SR2	A	B	C	-	-	-	A	B	C	A	B	C		
SR3	A	B	C	A	B	C	A	-	-	A	B	C		
SR4	A	B	-	A	B	C	A	B	C	A	B	C		
SR5	A	B	C	A	B	C	A	B	C	-	-	-	Central Grounds (CG)	36
SR6	A	B	C	A	B	C	A	B	C	A	B	C		
SR7	A	B	C	A	B	C	-	-	-	-	-	-		
SR8	-	-	-	A	B	C	A	B	C	A	B	C		
SR9	A	B	C	A	B	C	A	B	C	A	B	C	Eastern Ridge (ER)	48
SR10	A	B	C	A	B	C	A	B	C	A	B	C		
SR11	A	B	C	A	B	C	A	B	C	A	B	C		
SR12	A	B	C	A	B	C	A	B	C	A	B	C		
SR13	A	B	C	A	B	C	A	B	C	A	B	C	Southern Ground (SG)	45
SR14	A	B	C	A	B	C	A	B	C	A	B	C		
SR15	A	B	C	A	B	C	A	B	C	A	B	C		
SR16	A	B	C	A	B	C	-	-	-	A	B	C		
SR17	A	B	C	A	B	C	A	B	C	A	B	C	Western Ridge (WR)	46
SR18	A	B	C	A	B	C	A	B	C	-	B	-		
SR19	A	B	C	A	B	C	A	B	C	A	B	C		
SR20	A	B	C	A	B	C	A	B	C	A	B	C		
**TOTAL**	**56**			**57**			**52**			**52**				**217**

[1] A dash (-) indicates that samples were unavailable.

Table S2. Combinations of wine samples in blending (50:50) for A-TEEM analysis with 6 groups for vintage blending and 10 groups for sub-region blending of Northern Grounds (NG), Central Grounds (CG), Eastern Ridge (ER), and Southern Grounds (SG). S1–4 etc. indicate sample numbers for blends that were prepared in duplicate, with each analysed in duplicate (i.e., 4 spectra were recorded per sample).

	**Vintage blending**		
	**2021**	**2020**	**2019**
2018	S1–4	S13–16	S21–24
2019	S5–8	S17–20	
2020	S9–12		

	**Sub-region blending**			
	**WR**	**SG**	**ER**	**CG**
NG	S1–4	S17–20	S29–32	S37–40
CG	S5–8	S21–24	S33–36	
ER	S9–12	S25–28		
SG	S13–16			

Table S3. Percentage of wine in the blend of 2018 and 2021 vintages. Blended wine samples prepared in duplicate (i.e., S1 and S2, S3 and S4).

	**Blending percentage (% v/v)**	
	**S1 and S2**	**S3 and S4**
2018	95	90
2021	5	10

Table S4. Percentage of wine in the blend of Southern Grounds (SG) and Western Ridge (WR) sub-regions. Blended wine samples were prepared in duplicate (i.e., S1 and S2, S3 and S4).

	**Blending percentage (% v/v)**	
	**S1 and S2**	**S3 and S4**
SG	85	50
WR	15	50

Table S5. Confusion matrices showing the performance parameters of different cross-validated XGBDA models from multi-block data (EEM and absorbance) for (A) vintages (Figure 5a in the main paper), (B) subregions (Figure 5b), (C) blending between vintages (Figure 6a) and (D) blending between subregions (Figure 6b).

	Class	No.	Sensitivity %[1]	Specificity %[2]	Error %[3]	Precision %[4]	F1[5]
**A**	2018	112	100.00	100.00	0.00	100.00	1.00
	2019	112	100.00	100.00	0.00	100.00	1.00
	2020	104	100.00	100.00	0.00	100.00	1.00
	2021	104	100.00	100.00	0.00	100.00	1.00
**B**	1-NG	84	98.81	100.00	0.60	100.00	0.99
	2-CG	72	100.00	100.00	0.00	100.00	1.00
	3-ER	96	100.00	100.00	0.00	100.00	1.00
	4-SG	90	98.89	99.71	0.70	99.71	0.99
	5-WR	92	100.00	99.71	0.15	99.71	1.00
**C**	2018/2021	4	75.00	100.00	12.50	100.00	0.86
	2019/2021	4	100.00	100.00	0.00	100.00	1.00
	2020/2021	4	100.00	100.00	0.00	100.00	1.00
	2018/2020	4	100.00	100.00	0.00	100.00	1.00
	2019/2020	4	100.00	95.00	2.50	95.24	0.98
	2020/2019	4	100.00	100.00	0.00	100.00	1.00
**D**	A-NG/WR	4	100.00	97.22	1.39	97.30	0.99
	B-CG/WR	4	75.00	100.00	12.50	100.00	0.86
	C-ER/WR	4	100.00	100.00	0.00	100.00	1.00
	D-SG/WR	4	100.00	100.00	0.00	100.00	1.00
	E-NG/SG	4	100.00	97.22	1.39	97.30	0.99
	F-CG/SG	4	100.00	97.22	1.39	97.30	0.99
	G-ER/SG	4	100.00	100.00	0.00	100.00	1.00
	H-NG/ER	4	75.00	100.00	12.50	100.00	0.86
	I-CG/ER	4	75.00	100.00	12.50	100.00	0.86
	J-NG/CG	4	100.00	100.00	0.00	100.00	1.00

[1] Sensitivity %: proportion of positive cases that were correctly identified = 100 × TP/ (TP + FN)

[2] Specificity %: proportion of negative cases that were classified correctly = 100 × TN/ (TN + FP)

[3] Misclassification error %: proportion of samples which were incorrectly classified = 100×(1-accuracy), where accuracy = (TP + TN)/ (TP + TN + FP + FN)

[4] Precision %: proportion of positive cases giving a true positive result = 100 × TP/ (TP + FP)

[5] F1 Score: harmonic mean of Precision and Sensitivity = 2TP/ (2TP + FP + FN).

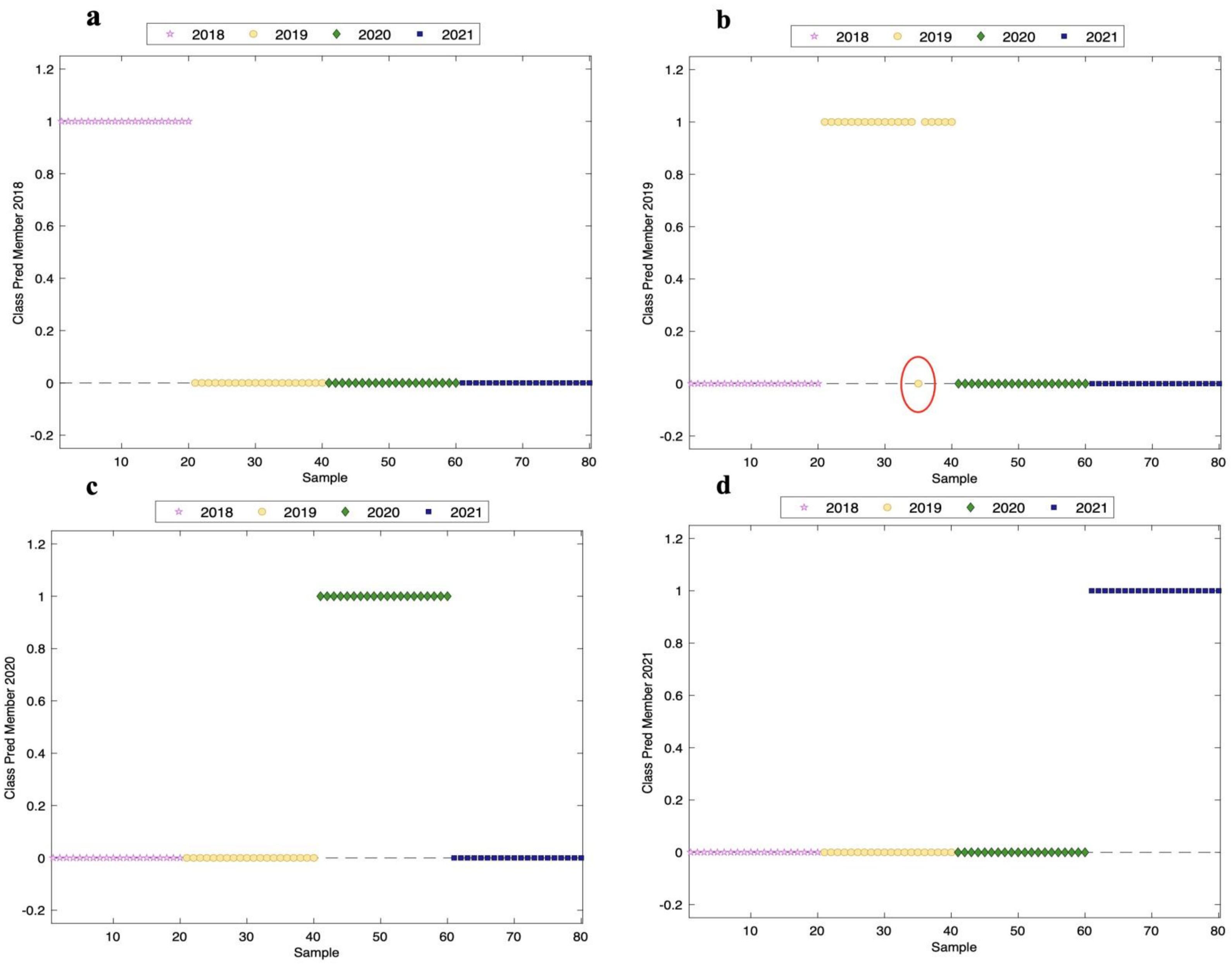

Figure S1. Class predicted member for test set samples (n = 80) from XGBDA modelling of multi-block A-TEEM data for vintage (a) 2018 (lilac), (b) 2019 (yellow), (c) 2020 (green), and (d) 2021 (blue). Sample outlined in red is misclassified.

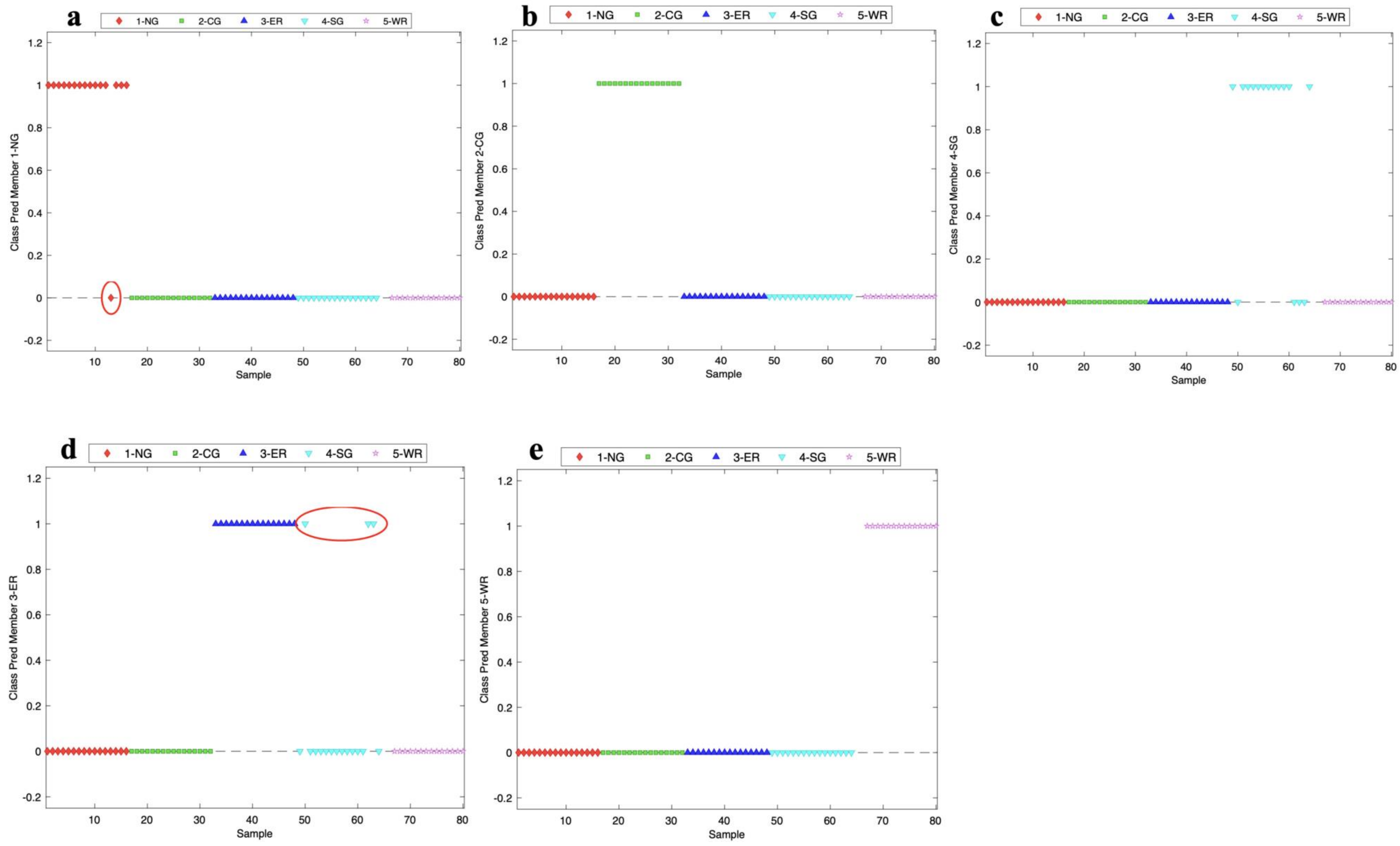

Figure S2. Class predicted member for test set samples (n = 80) from XGBDA modelling of multi-block A-TEEM data for subregion (a) Northern Grounds (NG), (b) Central Grounds (CG), (c) Southern Grounds (SG), (d) Eastern Ridge (ER) and (e) Western Ridge (WR). Samples outlined in red were misclassified data.

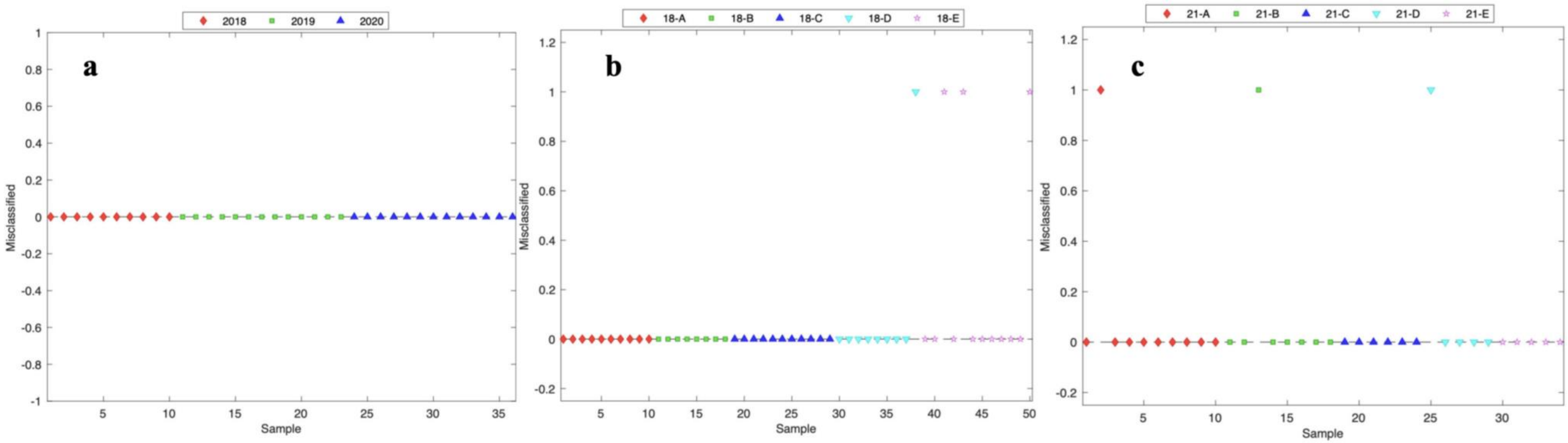

Figure S3. Class predicted member using the XGBDA model established by Ranaweera et al. (2023) with multi-block A-TEEM test data from the present study showing vintage (a) 2018 (red), 2019 (green), and 2020 (blue), and the five sub-regions for vintage (b) 2018 and (c) 2021. NG (A, red), CG (B, green), ER (C, dark blue), SG (D, light blue), WR (E, lilac).

References

Ranaweera, R. K. R., Bastian, S. E. P., Gilmore, A. M., Capone, D. L., & Jeffery, D. W. (2023). Absorbance-transmission and fluorescence excitation-emission matrix (A-TEEM) with multi-block data analysis and machine learning for accurate intraregional classification of Barossa Shiraz wine. *Food Control*, *144*, 109335. https:/doi.org/10.1016/j.foodcont.2022.109335.
